# Establishment and Morphological Characterization of Patient-Derived Organoids from Breast Cancer

**DOI:** 10.1186/s12575-019-0099-8

**Published:** 2019-06-15

**Authors:** S. Mazzucchelli, F. Piccotti, R. Allevi, M. Truffi, L. Sorrentino, L. Russo, M. Agozzino, L. Signati, A. Bonizzi, L. Villani, F. Corsi

**Affiliations:** 1 Nanomedicine Laboratory, Department of Biomedical and Clinical Sciences “Luigi Sacco”, University of Milan, via G. B. Grassi, 74, 20157 Milan, Italy; 2 Breast Unit, Istituti Clinici Scientifici Maugeri IRCCS, via S. Maugeri, 4, 27100 Pavia, Italy; 3 Pathology Unit, Istituti Clinici Scientifici Maugeri IRCCS, via S. Maugeri, 4, 27100 Pavia, Italy

**Keywords:** Breast Cancer, Patient-derived organoid, Biopsy

## Abstract

**Background:**

Patient-derived organoids (PDO) technology represents an emerging tool for the study of tumor biology and drug responsiveness, thus being useful to design personalized medicine approaches. Despite several studies and clinical trials are ongoing using PDO from colorectal and pancreatic cancer, only few research papers have been published exploiting PDO from breast cancer. Here, we have developed a new protocol to establish PDO from surgical and biopsy samples. Furthermore, we have set up also the methodologies adopted for culture and morphological evaluations.

**Results:**

Surgical and core biopsy specimens collected from 33 patients with diagnosis of breast cancer have been processed using the protocols here described obtaining PDO from cancerous and healthy mammary tissue (when available) in a quick and easy way with good yields. The more critical aspects influencing the yield were the characteristic of the tissue of origin (healthy vs tumor tissue) and the amount of material obtained after enzymatic digestion process. Success rate from healthy samples was about 20,83%, while this percentage was higher in samples from cancer tissue (i.e. 87,5%). Also the morphological characterization of breast cancer PDO by brightfield and transmission electron microscopy has been reported.

**Conclusions:**

Despite obtaining some organoids from a surgical or biopsy specimen is not a difficult procedure, the establishment of a stable organoid line able to grow and replicate, suitable for long-term biobank storage, is not so obvious. A novel, simple and quick procedure to obtain PDO from surgical and biopsy samples is here proposed to achieve high success rate .

## Background

An emerging tool in the study of tumor clonal evolution is represented by patient-derived organoids (PDO) [1]. PDO technology has been firstly employed to investigate colorectal cancer and to study tumor biology and the mechanisms of clonal evolution responsible for metastases formation [1–3]. Besides the possibility to increase knowledge about the molecular mechanisms related to tumor evolution, PDO have captured the researcher’s interest as they represent a useful tool in drug discovery and screening, paving the way for the development of novel approaches to personalized medicine [4]. Indeed, unlike the in vivo models of patient-derived xenograft (PDX), PDO promise to obtain an ex vivo real-time chemosensitivity evaluation for each patient’s tumor, which could be compatible with the timing of treatment, rapidly aiding the clinical decision-making [1, 4]. Some preclinical and clinical trials are ongoing using PDO derived from colorectal cancer samples like TUMOROID and SENSOR studies, but evidences with breast cancer are still poor. Recently, PDO have been generated from breast cancer demonstrating their capability to predict drug sensitivity and tumor clonal evolution, evidencing their huge potential as drug screening platform to identify optimal and patient-tailored treatments [5, 6].

However, at present only few papers describe methodologies to obtain breast cancer organoids from clinical samples. The first one is a work from Whelm group that describes in detail applications related to organoid technology [5]. It describes how to obtain PDO from clinical specimen such as surgical resections, biopsy samples and ascites effusions. Description of procedures is accurate, but the proposed methodology to separate organoids from single cells is difficult and time consuming: repeated centrifugation cycles are necessary to separate PDO from single cells and to enrich PDO fraction. Moreover, these protocols are not designed to establish a PDO culture for biobank purposes and the procedures of breast cancer organoid maintenance and amplification are not described in detail [5]. The second work is a research paper recently published by Clevers and coworkers that describe the generation of a biobank of breast cancer PDO, which perfectly recapitulates patients heterogeneity. The main novelties from this approach are that (i) the digestion is been performed only with collagenase and (ii) for a limited time (1–2 h at 37 °C) and (iii) there is not a separation between PDO and single cells obtained after the enzymatic digestion [6]. Here, we propose a new approach to obtain PDO from surgical and biopsy samples that integrates the most effective procedures from the two methods proposed by these research groups in a quick and simple way. Moreover, we display the methodologies adopted for PDO culturing and for further morphological evaluations by immunohistochemistry and transmission electron microscopy.

## Results

**Fig. 1:**
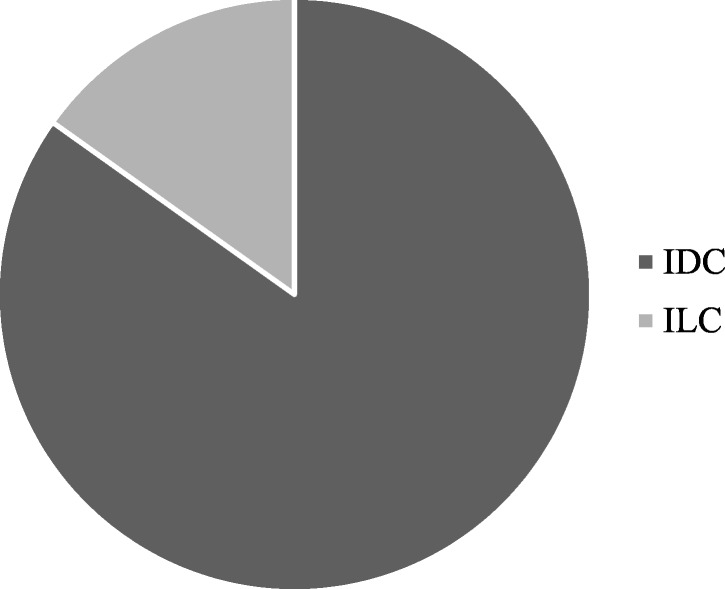
Graphical representation of distribution of histological subtypes of tumors from recruited patients

**Fig. 2:**
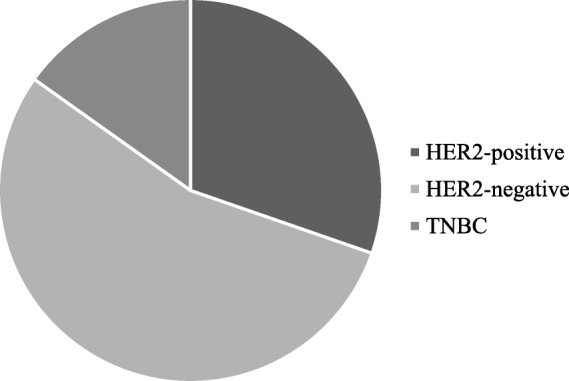
Graphical representation of distribution of membrane receptor immunohistochemistry in tumors from recruited patients

The aim of the present study is to develop a simple and quick method to obtain PDO from surgical and biopsy specimens of breast cancer patients, to assess the reliability of PDO in recapitulating breast cancer features for further translational studies. Using the methods described in this paper, we have processed surgical and core biopsy specimens collected from 33 patients treated at the Breast Unit of ICS Maugeri IRCCS (Pavia, Italy) from October 2018 to January 2019. All PDO have been stored in the “Bruno Boerci” biobank established by the “Bruno Boerci” foundation and hosted by ICS Maugeri IRCCS. Histological characterization of primary tumors revealed that 84,84% (28/33) were invasive ductal carcinoma (IDC), while 15,15% (5/33) were invasive lobular carcinoma (ILC) (Fig. 1). Immunohistochemistry assessment of receptors status evidenced that 47,62% (10/33) were HER2-positive, 85,71% (19/33) were HER2-negative and only 23,81% (5/33) were triple negative breast cancer (TNBC) (Fig. 2).

## Establishment of PDO from Surgical Samples

**Fig. 3:**
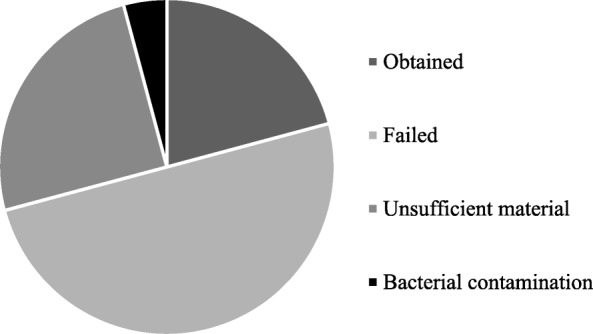
Graphical representation of results obtained applying protocol #1 in healthy samples from breast cancer surgical resection (HB). Specimen processed from HB could have been: obtained (Obtained), seeded but have not resulted in a PDO culture (Failed), not been seeded due the scarce amount of cells (Unsufficient material) or discarded due to bacterial contamination (bacterial contamination)

**Fig. 4:**
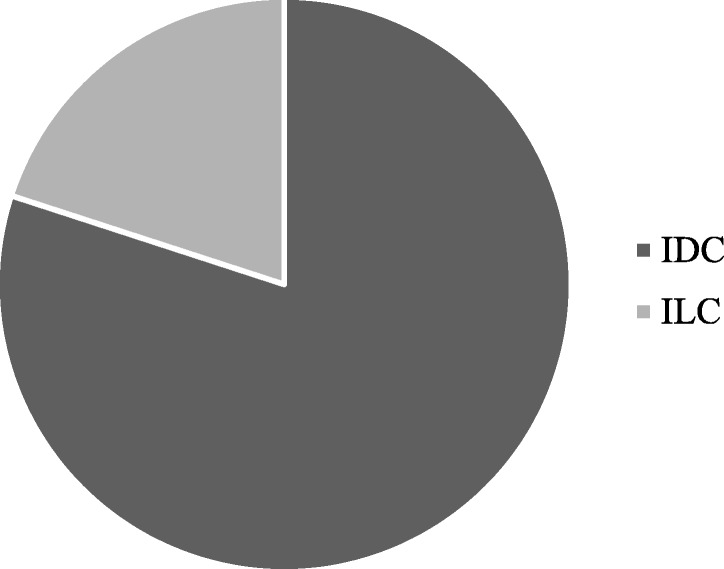
Graphical representation of histological classification of primary lesions HB that have allowed us to obtain a PDO culture applying protocol #1

The procedure accurately described in method section (protocol #1) consists of an overnight digestion at 37 °C with an enzymatic cocktail of collagenase III and hyaluronidase, followed by sequential filtrations on cell strainers with different porosity (100 and 20 μm) to separate organoids from single cells. In the reference period (from October 2018 to January 2019), we have processed surgical samples from 24 patients. All included patients were affected by breast cancer at any stage with 79,16% (19/24) of them classified as patients with IDC, while 20,833% (5/24) as ILC. For each of them, we have collected and processed one tumor sample (TB) and one healthy sample from the most clear margin (HB) as assessed by pathologic gross examination. About 50,00% (12/24; failed) of specimens processed from HB has been seeded but has not resulted in a PDO culture, 25,00% (6/24; unsufficient material) has not been seeded due the scarce amount of cells and 4,16% (1/24; bacterial contamination) has been thrown away, due to bacterial contaminations observed during handling. A PDO culture has been obtained from HB resection only in 5 of 24 cases, allowing to obtain a success yield of 20,83% (Fig. 3). Histologic analysis of the sample from which these 5 PDO were derived revealed that 80% (4/5) were classified as IDC, while 20% (1/5) were ILC (Fig. 4). Moreover, all the PDO (5/5) obtained from HB samples belonged to HER2-negative primary lesions by immunohistochemistry.

**Fig. 5:**
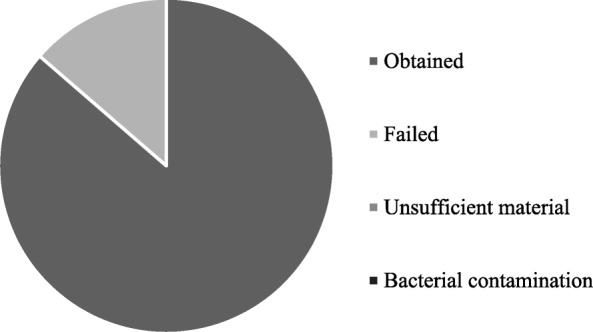
Graphical representation of results obtained applying protocol #1 in surgical resection defined by gross examination of the pathologist as tumor tissue (TB). Specimen processed from TB could have been: obtained (Obtained), seeded but have not resulted in a PDO culture (Failed), not been seeded due the scarce amount of cells (Unsufficient material) or discarded due to bacterial contamination (bacterial contamination)

**Fig. 6:**
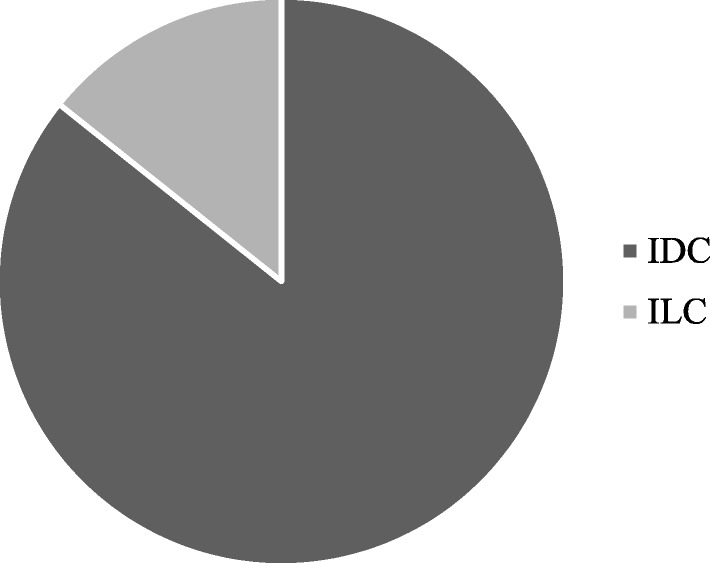
Graphical representation of histological classification of primary lesions that have allowed us to obtain a TB-PDO culture applying protocol #1

**Fig. 7:**
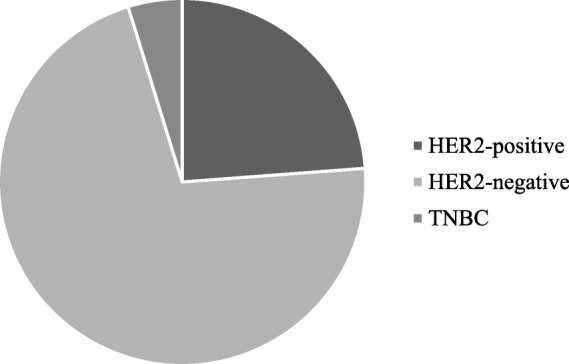
Graphical representation of membrane receptor classification of primary lesions TB that have allowed us to obtain a PDO culture applying protocol #1

About TB samples, 12,5% (3/24) of processed specimens has not resulted in a PDO culture, while none has been excluded before seeding for lack of PDO in processed sample or discarded due to bacterial contamination. A PDO culture has been obtained from TB resection in 21 of 24 cases, allowing to obtain a success yield of 87,5%. Moreover, 2 of 21 PDO obtained from TB have been frozen (protocol #6) after a culture period of 2 months and stored in nitrogen gas for Biobank purposes (Fig. 5). Histologic analysis of TB primary lesions, which have resulted in PDO culture, revealed that 85,71% (18/21) were IDC, while 14,28% (3/21) were ILC (Fig. 6). Moreover, 71,42% (15/21) of PDO obtained from TB samples belonged to primary lesions of HER2-negative tumors, while 23,81% (7/21) were from HER2-positive and 4,76% (1/21) Triple Negative Breast cancer (TNBC). (Fig. 7).

## Establishment of PDO from Biopsies

**Fig. 8:**
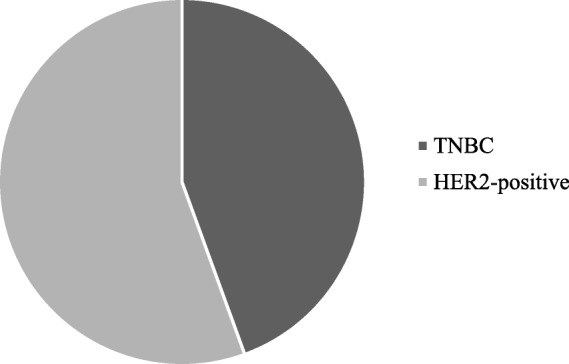
Graphical representation of receptor status of biopsy samples successfull in PDO culture applying protocol #2

In the same period, we have collected core biopsy samples from further 9 patients affected by breast cancer with indication for neoadjuvant chemotherapy (locally advanced or node-positive disease and/or HER2-positive or triple-negative subtype). After obtaining the informed consent, one tissue sample for PDO establishment was obtained from each patient during standard core biopsy needed for lesion characterization before starting neoadjuvant treatment. In 100% (9/9) of processed specimens we have obtained enough material to be seeded in a well of 24-wells plate. Histologic analysis revealed that 100% (9/9) of original biopsies were classified as IDC, with 55,55% (5/9) of them belonging to HER2-positive subtype, while 44,44% (4/9) to TNBC (Fig. 8).

## Morphological Characterization of PDO

**Fig. 9:**
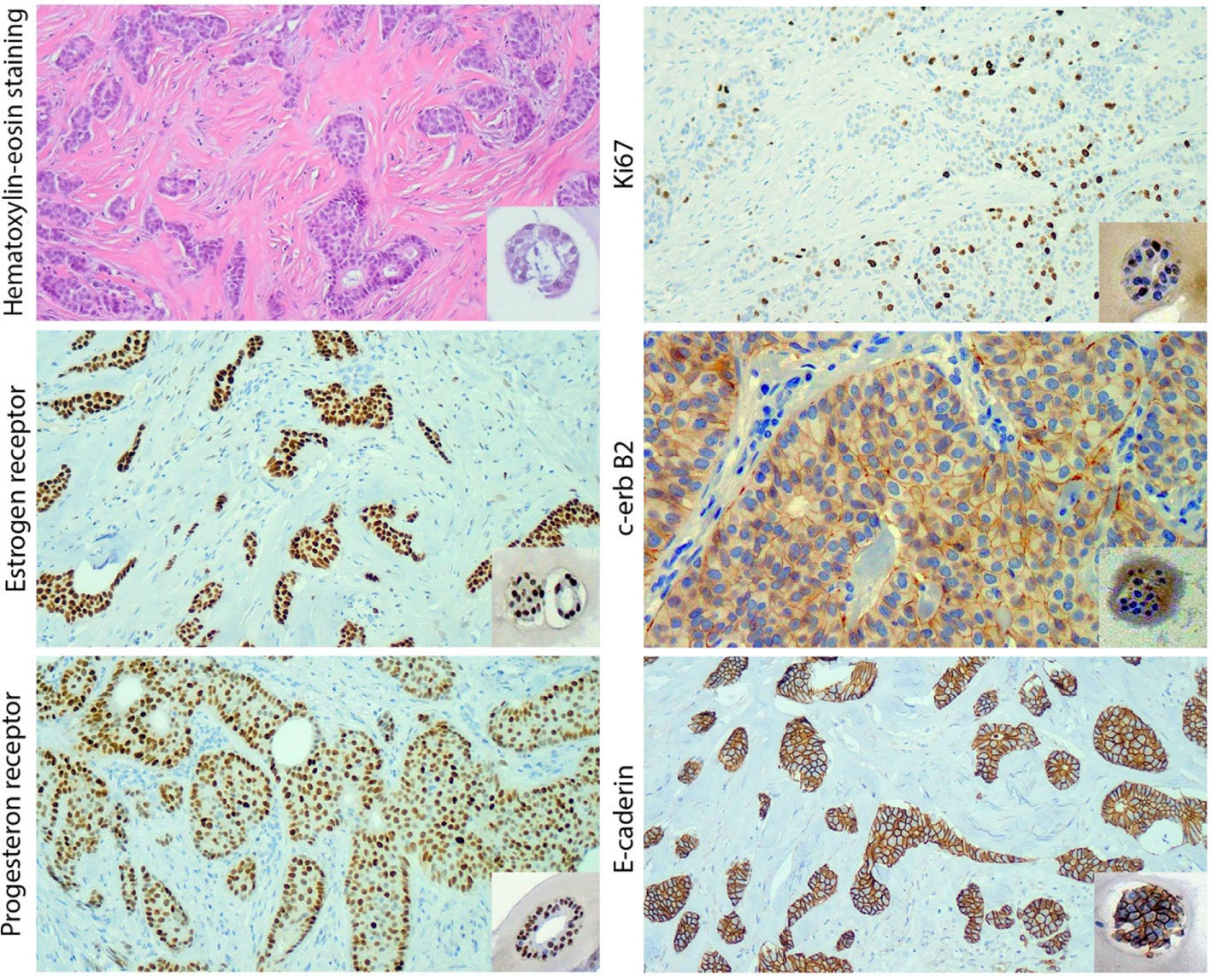
Hematoxylin and eosin staining and immunohistochemistry (ICH) labelling performed on surgical tissue of origin and derived PDO. ICHs have been performed to label ki67, c-erb B2, E- caderin, estrogen and progesterone receptor. Surgical tissue of origin is represented in large images, while PDOs are in inserts

In order to characterize PDO morphology and histology, we have set up protocols for immunohistochemistry (ICH) and transmission electron microscopy analysis. For ICH, the matrigel droplet containing PDO has been removed from the culture plate with a sterile lifter and embedded in an agarose matrix before formalin fixation. Then, paraffin embedding and ICH reactions have been performed following the procedures used for samples of clinical routine in order to allow an optimal comparison. Indeed, the critical feature of the emerging PDO models is the demonstration of concordance with patient histology. Results displayed in Fig. 9 evidenced a good degree of concordance between patient’s tumor and PDO histology.

**Fig. 10:**
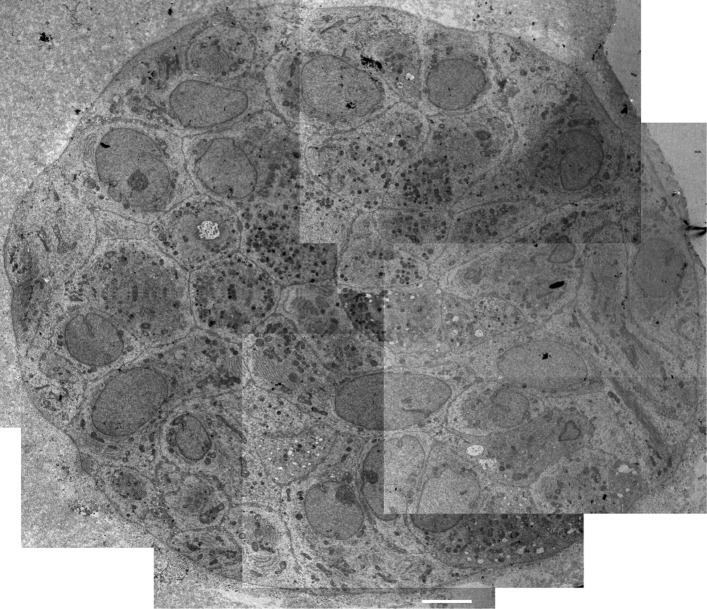
Mosaic reconstitution of Transmission Electron Microscopy of a PDO. Scale bar = 5 μm. Images acquired with Tecnai Spirit BT (FEI) transmission electron microscope, magnification of each single image 2550 ×

**Fig. 11:**
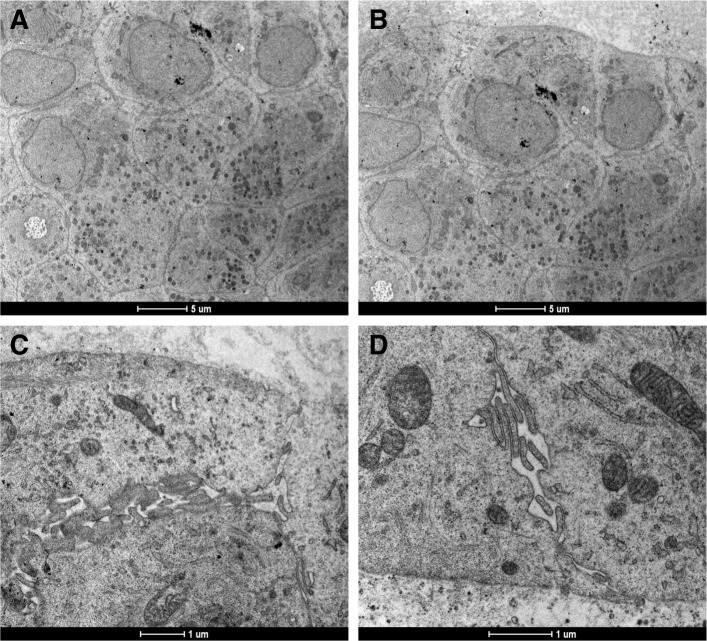
Transmission Electron Microscopy images of typical specialization and characteristic features in a PDO. In panels **a** and **b** it is possible to appreciate the presence of intracellular granules, while in panels **c** and **d** we observe structures similar to intercellular channels. Image acquired with Tecnai Spirit BT (FEI) transmission electron microscope. Panel **a** and **b** magnification 2250×; panel **c** magnification 20,500× and panel **d** magnification 26,500 ×

Transmission electron microscopy imaging has been performed following standard preparation of the sample. In detail, a single drop of PDO embedded in matrigel has been removed from the culture plate with a sterile lifter, fixed with buffered glutaraldehyde, dehydrated, stained and embedded in epon. The sample has been cut in appropriate slices and analyzed to obtain a mosaic reconstitution of the entire PDO. This approach allows to appreciate the structural organization of PDO and the presence of regions with typical specialization and characteristic features (Figs. 10 and 11).

## Discussion

Obtaining some organoids from a surgical or biopsy specimen is not a difficult procedure. Indeed, even if we were not able to see a cellular pellet after the digestion procedure, when we seeded it and we put the Matrigel drop under light microscope we could identify some PDO in almost all cases. Despite this, the establishment of a PDO line able to grow and replicate in culture, as suitable for a biobank storage, is not so easy. It is necessary to acquire self-confidence with 3D culture procedures, but also with each line. Indeed, PDO with compact spherical morphology should be treated in harsh condition to obtain a successful shearing. However, a mild mechanical shearing is sufficient for PDO with grape-like or non-compact morphology in order to obtain organoids fragmentation. TryPLE shearing may convert a big PDO in a single cell suspension in a little amount of time, so it is necessary to pay maximum attention during this process. In particular, if the population is highly heterogenous in size, it is very crucial to take prompt and right decisions about shearing. Indeed, performing a TryPLE shearing on small PDO may result in the loss of culture if they split into single cells. On the other hand, waiting too long before shearing could risk to let the biggest organoids die. Therefore, an appropriate training and observation period are fundamental to tune cell culture technicians into PDO culture and to acquire the necessary confidence in decision making. Indeed, since they derive from specific tumors from unique patients, each PDO differs from another one and requires to adjust culturing time and shearing procedures. Continuous and thorough observation of organoids in culture is key to fine-tune knowledge of PDO features and morphology to take optimal decisions in order to culture them in the best way.

Moreover, if the starting material is too little, the probability to discard the PDO culture after a short time is very high, being organoids seeded at low density in a single well not able to grow well. In addition, we should also consider that different success rates could be obtained in case we process cancer samples (TB) or healthy tissue (HB), as expected since cancer cells are more proliferative than healthy ones. However, obtaining PDO also from healthy tissue is very important for further applications to allow a comparison with a control.

These issues coupled with the high costs of Matrigel, culture medium and supplements have forced us to hypothesize some check points before seeding the digested sample. First of all, if the surgical specimen is from HB tissue and if it results in an invisible pellet after digestion, it could be better deciding to discard it without trying to establish a PDO culture, since the probability to success is too low. Indeed, we have observed that all the PDO from HB sample that have been discarded, were first seeded in a single well of 24-wells plate since the starting material was poor. Moreover, if the PDO culture grows slowly and it has not been expanded 2 months after initial processing, it becomes reasonable and more economically advantageous to stop its culturing. Therefore, the critical point in PDO establishment is to identify appropriate check points to verify whether each PDO culture is promising for biobank purposes.

## Conclusions

Despite obtaining PDO is not a difficult procedure, the establishment of an organoid line able to grow and replicate, suitable for a biobank storage, is not so easy. The present study reports a novel protocol to obtain PDO from breast cancer, either surgical specimens or biopsy. The proposed methodology yielded a good success rate in establishing PDO, which showed histological and biomolecular features of concordance with primary tumors, paving the way for ex vivo characterization of primary tumors to adequately and timely plan treatments. Given the promising results obtained with the protocol here presented, it becomes mandatory to enlarge the patients cohort to establish breast PDO and perform further studies.

## Methods

### Protocol #1: Tissue digestion from breast cancer surgical specimen

In the reference period, fresh tumor specimens from surgical resections have been collected from 24 patients affected by breast cancer at any stage and candidated to immediate breast-conserving surgery or total mastectomy. A written and oral informed consent to participate in the present protocol was obtained from each patient. Tumor specimens obtained from surgical resection could be stored at 4 °C in working medium (WM) until processing. Processing into organoids should be performed in sterile conditions within 48 h from the day of collection. All tissue processing was performed in a biosafety cabinet (hood) using Biosafety Level 2 (BSL2) techniques. The amount of tissue used for this procedure ranged from 0.17 g to 13.1 g.

**Fig. 12:**
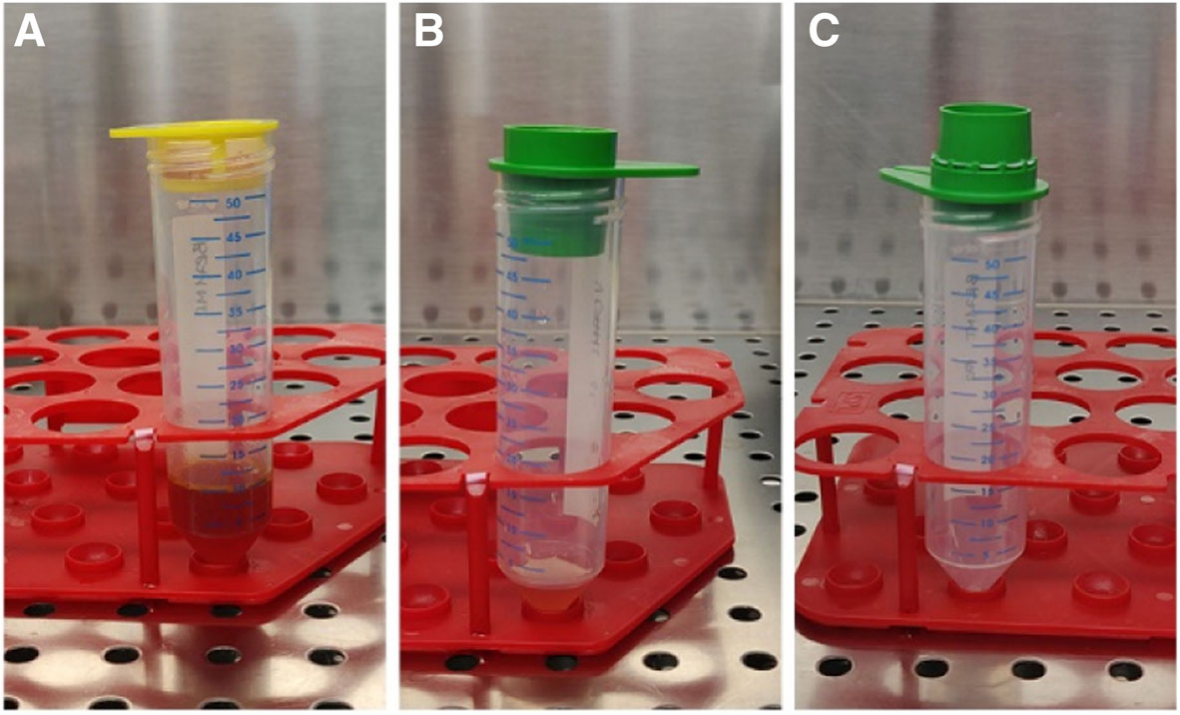
Images of the filtration passages to obtain PDO from a surgical specimen. **a** Filtration throught 100 μm cell strainer. **b** Entrapment of organoids on top of a 20 μm cell strainer and **c** recovery of organoids from the top of a 20 μm cell strainer

1. Transfer the solid tumor tissue in a 6 or 10 cm petri dish. Assure to keep the tissue moist with PBS.
2. Remove fat tissue and mince tumor sample with scalpel by criss cross motion.
3. Transfer the minced tissue in a 50 mL tube using a cell lifter and measure the net weight of the obtained tissue.
4. Digest the minced tissue in digestion buffer (10 mL/g of tissue; DMEM-F/12 supplemented with 10 mM HEPES, 2% BSA, 0.5 μg/ml hydrocortisone, 50 μg/mL gentamycin) supplemented with Collagenase III (Wortington #LS004182; 0.2 mg/mL) and with Hyaluronidase (Sigma #H3884; 1000 U/mL) for 16 h at 37 °C under shaking (200 rpm).
5. To remove undigested fragments and debris, filter on sterile 100 μm cell strainer and collect the flow-through sample (Fig. 12a). This medium mainly contains PDO, cancer single cells, red blood cells and fat.
6. Place a sterile 20 μm cell strainer on a new 50 mL sterile tube and label the tube as single cells fraction.
7. Discard 100 μm cell strainer and load the medium collected at passage 5 on the 20 μm cell strainer prepared in the previous step to separate single cells from organoids (Fig. 12b). After this passage organoids should be entrapped in the 20 μm cell strainer, while single cells should be collected in the tube.
8. Invert the 20 μm cell strainer on a new 50 mL tube and wash twice with 5 mL of WM to collect tumor organoids entrapped inside the 20 μm grid (Fig. 12c).
9. Discard the 20 μm cell strainer and collect organoids by centrifuging 5 min at 500×g, T = 8 °C.
10. Remove the supernatant avoiding to disrupt organoid pellet.
11. If the pellet contains red blood cells (observed as a red layer on top of the pellet), add 1 mL of TAC buffer and incubate 10 min in a 37 °C water bath. Then, neutralize with 10 mL of WM and centrifuge (5 min at 500×g, T = 8 °C) to collect organoids.
12. If the pellet contains a lot of fat, perform one or two additional washing step with 10 mL of WM.
13. Remove carefully the supernatant and resuspend organoid pellet in Matrigel 90% in WM (Corning, Matrigel Basement Membrane Matrix Growth Factor Reduced, Phenol Red Free, #356231) for seeding. Table 1 reports the volume of Matrigel necessary to seed organoids in plates of different formats. If the pellet is invisible, we advise to seed the sample in a well of 24-wells plate.
14. Seed in a pre-warmed multiwell plate, then invert the plate to avoid organoid sinking. After 10 min, transfer the seeded plate into the incubator for 20 min, to allow Matrigel solidification.
15. In the meantime, prepare Complete Culture Medium (CCM) [6]. At the end of the process of Matrigel solidification, invert the plate, gently add CCM to organoid-matrigel drops and transfer into incubator for culturing (refer to Table 2 for volume of CCM required/plate size).

**Table 1:** The volume of 90% Matrigel necessary to resuspend PDO before seeding in different plate format, established as μL /well

Plate format	μL of 90% Matrigel/well
6-wells	150 μL
12-wells	70 μL
24-wells	35 μL

**Table 2:** Summarizes the the volume of CCM required for each type of well

Plate format	mL of CCM/well
6-wells	1.5–2 mL
12-wells	1 mL
24-wells	0.5 mL

### Protocol #2: Tissue digestion from breast cancer biopsy

Fresh tumor biopsy of about 1.5 mm × 20 mm × 1.5 mm (2 samples obtained from a 12 gauge core needle biopsy) should be transferred into sterile WM and within 1 h from collection processed within 1 h from collection in sterile conditions in a biosafety cabinet (hood) using Biosafety Level 2 (BSL2) techniques.

1. Transfer the solid tumor tissue in a 6 cm petri dish and assure to keep the tissue moist with PBS.
2. Mince the tissue with scalpel by performing a criss cross motion.
3. Transfer the minced tissue in a pre-weighted 15 mL tube using a cell lifter.
4. Digest the minced tissue in 2 mL of digestion buffer (DMEM-F/12 supplemented with 10 mM HEPES, 2% BSA, 0.5 μg/ml hydrocortisone, 50 μg/mL gentamycin) supplemented with Collagenase III (Wortington #LS004182; 0.2 mg/mL) and with Hyaluronidase (Sigma #H3884; 1000 U/mL) for 3–4 h at 37 °C under shaking (200 rpm).
5. To remove undigested fragments and debris, filter on 100 μm cell strainer and collect the floe-through sample.
6. Wash the strainer with 5 mL WM.
7. Repeat step 6.
8. Collect organoids by 5 min at 500×g, T = 8 °C centrifugation.
9. Remove the supernatant avoiding to disrupt organoid pellet.
10. If the pellet contains red blood cells (observed as a red layer on top of the pellet), resuspend the pellet in 1 mL of TAC buffer and incubate 10 min in a 37 °C water bath. Then, neutralize with 10 mL of WM and centrifuge (5 min at 500×g, T = 8 °C) to collect organoids.
11. If the pellet contains a lot of fat, perform an additional washing step with 10 mL of WM.
12. Remove carefully the supernatant and resuspend organoid pellet in Matrigel 90% in WM (Corning, Matrigel Basement Membrane Matrix Growth Factor Reduced, Phenol Red Free, #356231) for seeding. In Table 1 is reported the volume of Matrigel necessary to seed organoids in plates of different formats. If the pellet is invisible, we advise to seed the sample in a well of 24-wells plate.
13. Seed in a pre-warmed multiwell plate, then invert the plate to avoid organoid sinking. After 10 min, transfer the seeded plate into the incubator for 20 min, to allow Matrigel solidification.
14. In the meantime, prepare CCM. At the end of the process of Matrigel solidification, gently add CCM to organoid-matrigel drops and transfer the plate back to the incubator for culturing (refer to Table 2 for CCM volume required/plate size).

### Protocol #3: PDO culturing

**Fig. 13:**
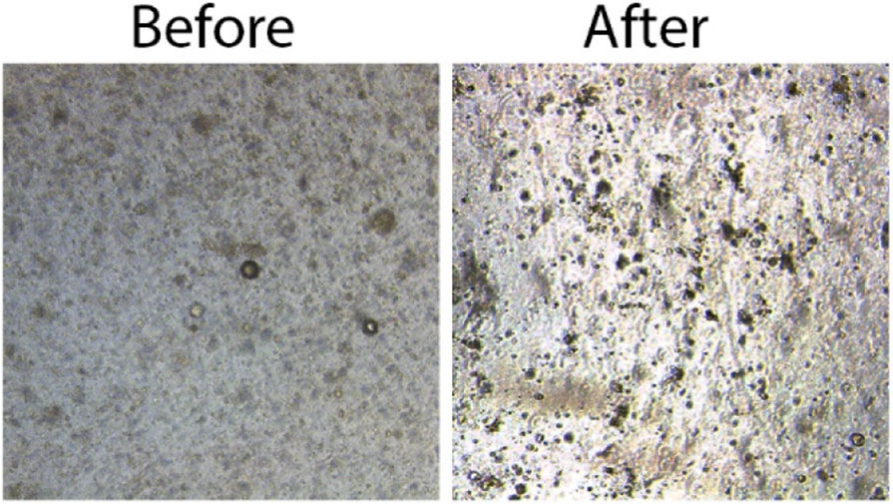
Images of PDO culture before and after expansion

**Fig. 14:**
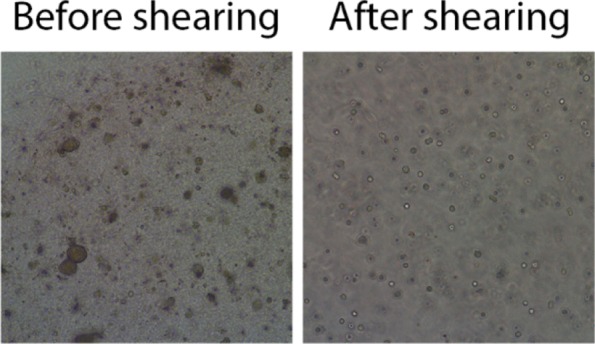
Images of PDO culture before and after the shearing process

**Fig. 15:**
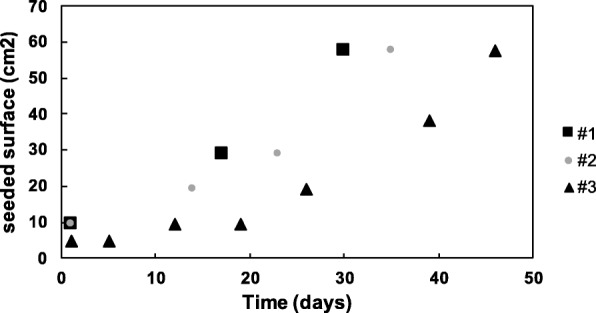
Schematic growth rate profile of three representative PDO cultures

Once seeded in matrigel droplets, generally, Breast Cancer organoids should be passed every 2 weeks to prevent that they become too much (a) or too big (b). Two different procedures should be performed in each case. The procedure (a) should be followed when density in Matrigel drop is high but organoids are small (Fig. 13); the protocol (b) should be performed when organoids are big while not necessary too crowded (Fig. 14). An example of organoids growth rate is shown in Fig. 15.

- Organoid expansion

1. Using CCM remained in the plate, dissolve the Matrigel drop and transfer the medium containing Matrigel and organoids in a new 15 mL tube.
2. Wash twice the plate using fresh WM (at least 2 mL to wash a well of 6 multiwell plate)
3. Collect washes and centrifuge 5 min at 500×g, T = 8 °C.
4. Discard the supernatant.
5. Resuspend in the appropriate amount of 90% Matrigel to expand the culture 1:2–1:3.
6. Seed organoids-matrigel drops and invert the plate to avoid organoid sinking.
7. After 10 min, transfer the seeded plate into the incubator for 20 min, to allow Matrigel solidification.
8. In the meantime, prepare Complete CCM.
9. At the end of the process of Matrigel solidification, gently add CCM to organoid-matrigel drops and transfer into incubator for culturing (Fig. 13).

b) Organoid shearing

PDO from breast cancer are generally compact organoids, therefore we suggest to follow the procedure described below. Instead, in case of grape-like or non compact organoids, avoid the use of TrypLE and divide PDO by mechanical shearing only.

1. Using CCM remained in the plate, dissolve the Matrigel drop and transfer the medium containing Matrigel and organoids in a new 15 mL tube.
2. Wash twice the plate using fresh WM (at least 2 mL to wash a well of 6 multiwell plate)
3. Collect washes and centrifuge 5 min at 500×g, T = 8 °C.
4. Discard the supernatant leaving 1 mL of WM.
5. Add 1 mL of TrypLE to obtain a WM:TrypLE ratio of 1:1
6. Using a flamed glass Pasteur, pipette up-and down for 20 times organoid suspension to combine enzymatic shearing of TrypLE with mechanical shearing.
7. Check shearing process under the light microscope. If there aren’t signs of organoid rupture continue mechanic shearing pipetting up and down 10–15 times more.
8. Neutralize TrypLE with 10 mL of WM and centrifuge to collect sheared organoids.
9. Discard the supernatant and resuspend organoids in the appropriate amount of 90% Matrigel to expand the culture 1:2–1:3.
10. Seed organoids-matrigel drops and invert the plate to avoid organoid sinking.
11. After 10 min, transfer the seeded plate into the incubator for 20 min, to allow Matrigel solidification.
12. In the meantime, prepare CCM.
13. At the end of the process of Matrigel solidification, gently add CCM to organoid-matrigel drops and transfer into incubator for culturing (Fig. 14).

### Protocol #4: PDO paraffin embedding for immunohistochemistry and histology

**Fig. 16:**
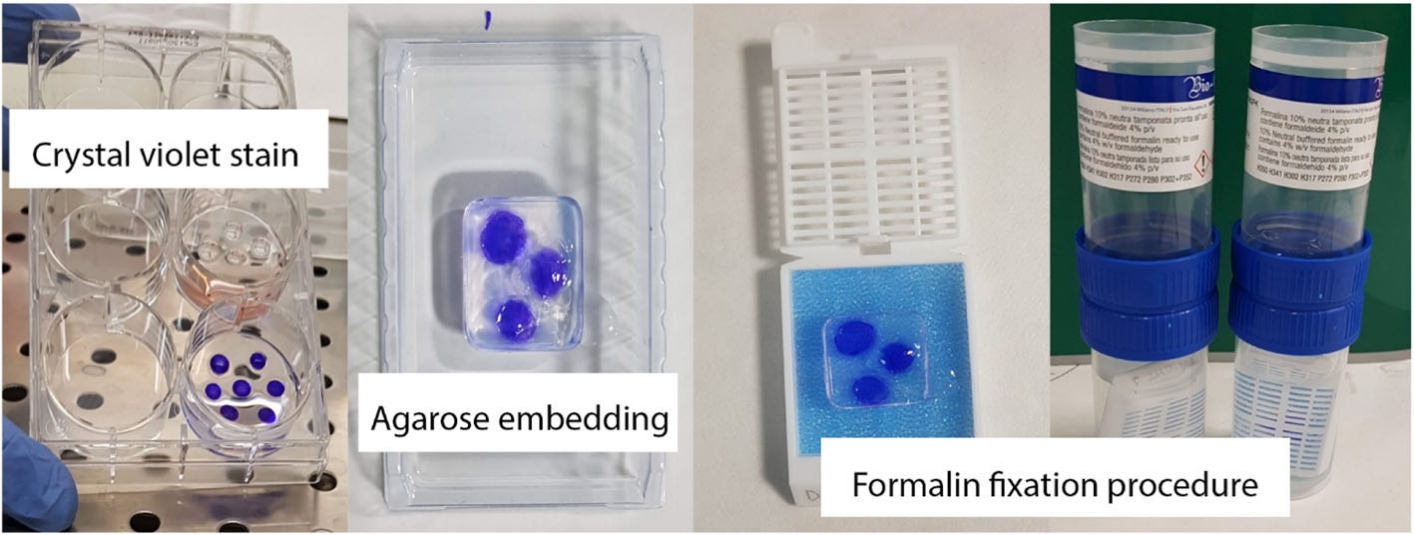
Images of main steps to manage PDO to obtain Formalin-Fixed and Paraffin Embedded sample. The procedure to prepare PDO for paraffin embedding could be summarized into 3 main steps: 1) crystal violet stain; 2) agarose embedding and 3) formalin fixation after the placement of the agarose cube containing PDOs in the histological box

1. Remove CCM from the selected plate.
2. To easily visualize and manipulate PDO drop label it by 30 min incubation with 0.05% crystal violet.
3. At the end of incubation wash PDO thrice with PBS to remove the excess of crystal violet (Fig. 16).
4. At the same time, dissolve agarose in milliQ water boiling the mixture to obtain a suspension of 2% agarose.
5. Remove a labelled drop of PDO with a cell lifter from the multi well plate and place it in a 15 × 15 × 5 mm mold pre-filled with unsolidified agarose 2%.
6. Place inside the agarose matrix about 3–4 Matrigel drops of PDO.
7. Wait about 15 min to obtain complete solidification of 2% agarose matrix (Fig. 16).
8. Remove the agarose piece from the mold and place it in a histological cassette in an oriented way (Fig. 16).
9. Put the histological cassette in 10% buffered formalin for fixation (Fig. 16).
10. Fixation and paraffin embedding have been performed using histo star embedding workstation (ASP300 Leica). After 2 h of incubation in buffered Formalin 10%, the sample was dehydrated with 3 cycles of 1 h and one of 2 h in 95% ethanol, followed by 2 cycles of 1 h and one of 2 h in absolute ethanol. Then, the sample was subjected to 2 cycles of incubation in Bioclear (Bio-Optica) for 1 h each, followed by one cycle lasting 2 h. After that, the sample was included in paraffin wax. Inclusion has been performed by two cycle of 1 h followed by one of 2 h.
11. Cut paraffin-embedded PDO in histological slides of 3,5 μm and labelled with VENTANA, Bench Mark ULTRA following automatized standard protocols established for E-caderin, c-ErbB2, estrogen receptor, progesterone receptor and Ki67 immunohistochemistry (ICH). Clones of antibodies used for immunohistochemistry are reported in Table 3.
12. At the end of ICH procedure, counter stain histological slides with eosin, dehydrate with ethanol (2 cycles in 95% ethanol and 2 cycles in 100% ethanol) and Bioclear and mount with a coverslip.

**Table 3:** Summarizes the antibodies used for ICH with VENTANA

Target	Commercial name	clone	Product code
c-erbB2	PATHWAY anti-HER2/neu	4B5 Rabbit Mab	05278368001
E-caderin	E-caderin	EP700Y Rabbit Mab	05973872001
Ki67	CONFIRM anti-Ki67	30–9 Rabbit Mab	05278384001
Estrogen Receptor	CONFIRM anti-ER	SP1 Rabbit Mab	05278406001
Progesteron Receptor	CONFIRM anti-ER	1E2 Rabbit Mab	05277990001

### Protocol #5: PDO processing for TEM imaging

1. Remove a drop of PDO embedded in 90% Matrigel with a cell lifter from a multi well plate and place it in a 1.5 mL tube.
2. Add 1 mL of 2.5% glutaraldehyde in cacodylate buffer overnight.
3. Wash thrice with cacodylate buffer.
4. Fix with osmium tetroxide 1.5% in cacodylate buffer for 2 h.
5. Wash again with cacodylate buffer.
6. Dehydrate with an ascending ladder of ethanol from 50 to 100%.
7. Include in epon.
8. Cut slides of 70–80 nm and label them with uranil acetate and lead citrate.
9. Acquire images with the Transmission Electron Microscope Tecnai Spirit BT FEI.

### Protocol #6: PDO freezing

Once obtained a 6 well plate full of organoids ranging from 40 to 100 μm, we could proceed to freezing. 2–3 days before freezing, one should share them in order to obtain a pool of dense and homogeneously small organoids.

1. Using CCM remained in the plate, dissolve the Matrigel drop and transfer the medium containing Matrigel and organoids in a new 15 mL tube.
2. Wash twice the plate using fresh WM (at least 2 mL to wash a well of 6 multiwell plate)
3. Collect washes and centrifuge 5 min at 500×g, T = 8 °C.
4. Discard the supernatant leaving 1 mL of WM.
5. Add 1 mL of TrypLE to obtain a WM:TrypLE ratio of 1:1.
6. Using a flamed glass Pasteur, pipette up-and down for 20 times organoid suspension to combine enzymatic shearing of TrypLE with mechanic shearing.
7. Check shearing process under the light microscope. If there aren’t signs of organoid rupture continue mechanic shearing pipetting up and down 10–15 times more.
8. Neutralize TrypLE with 10 mL of WM and centrifuge to collect sheared organoids.
9. Discard the supernatant and resuspend organoids in the appropriate amount of 90% Matrigel to re-seed them in a 6 multiwell plate.
10. Seed organoids-matrigel drops and invert the plate to avoid organoid sinking.
11. After 10 min, transfer the seeded plate into the incubator for 20 min, to allow Matrigel solidification.
12. In the meantime, prepare Complete Culture Medium (CCM).
13. After Matrigel solidification, gently add CCM to organoid-matrigel drops and transfer the plate into the incubator for culturing.
14. After a couple of day collect organoids using CCM in the plate to dissolve the Matrigel drop and transfer the medium containing Matrigel and organoids in a new pre-chilled 15 mL tube. Use two 15 mL tubes to collect organoids from a full 6 multiwell plate.
15. Leave the tube on ice and wash twice the plate using Ice-cooled WM (at least 2 mL to wash a well of 6 multiwell plate)
16. Collect washes and centrifuge 5 min at 500×g, T = 8 °C.
17. Discard the supernatant leaving 1 mL of WM.
18. Add 11 mL of ice-cooled WM and incubate 10 min in ice. This incubation should help in dissolve Matrigel.
19. Leaving tubes in ice, pipet up and down and centrifuge 5 min at 500×g, T = 8 °C.
20. Discard the supernatant and repeat the washing 18–20 if the suspension of Matrigel and organoids is more than 0.2 mL.
21. Leave the tubes in ice and add 3 mL of freezing medium in each 15 mL tube.
22. Prepare 6 aliquots of PDO suspension from each 15 mL tube by pipetting 0.5 mL of PDO suspension into each cryovial.
23. Immediately transfer cryovials in a Nalgene cryostep for 24 h at − 80 °C, then store the aliquots in liquid nitrogen for long term conservation.

### Protocol #7: PDO thawing

The day before the thawing of an organoid vial it is necessary to pre-warm a 24-well tissue culture plate in the incubator.

1. Pre-warm WM at 37 °C before use.
2. Thaw the vial rapidly by soaking in a 37 °C water bath under agitation until there is a piece of frozen material left.
3. Remove the vial from the water bath and clean it with ethanol before the transfer in a biosafety hood.
4. Transfer the thawed organoids to a sterile 15 ml falcon tube and add 1 ml of warm WM drop by drop while shaking the bottom of the tube.
5. Mix carefully by pipetting up and down a few times to dilute the freezing medium and slowly add 9 mL of warm WM into the 15 mL tube containing the organoids.
6. Invert the tube a few times.
7. Centrifuge 5 min at 500×g, T = 8 °C
8. Discard the supernatant without disrupting the pellet and resuspend the pellet in the same volume of 90% matrigel that was frozen in the vial (about 70 μL).
9. Seed crowded in a pre-warmed 24-multiwell plate to support organoids recovery from thawing procedure.
10. Invert the plate to avoid organoid sinking.
11. After 10 min, transfer the seeded plate into the incubator for 20 min, to allow Matrigel solidification.
12. In the meantime, prepare CCM.
13. After Matrigel solidification, gently add CCM to organoid-matrigel drops and transfer into incubator for culturing.
14. Follow organoids growth for some day to verify that the freezing/thawing process has been placed correctly.

#### Materials

Human breast primary tumor tissue from surgical specimen or from biopsy (fresh; the quantity usually varies).

Ice

6 cm Ø Petri dish, sterile (Euroclone, #ET2060).

10 cm Ø Petri dish, sterile (Euroclone, #ET2100).

Nalgene Cryo 1 °C freezing container filled with isopropanol (Nalgene #5100–0001).

50 mL conical tubes, sterile (Euroclone, #ET5050B).

15 mL conical tubes, sterile (Euroclone, #ET5015B).

2 mL cryovial tubes, sterile (Euroclone, #ECC3112SS).

24-well standard tissue culture plate (Euroclone, #ET3024).

12-well standard tissue culture plate (Euroclone, #ET3012).

6-well standard tissue culture plate (Euroclone, ET#3006).

Disposable scalpels (#10 blades), razor blades, sterile.

Disposable cell lifter (Fisher #08–100-240), sterile.

Cell strainer (100 μm, Pluristrainer, #43–50,100-51), sterile.

Cell strainer (20 μm, Pluristrainer, #43–50,020-03), sterile.

Parafilm M.

5 mL tips (Euroclone, #EPS05N).

10 mL tips (Euroclone, #EPS10N).

P1000 low retention filter tips (ClearLine,#713,119).

P200 low retention filter tips (ClearLine, #713131).

P10 tips (Sorenson, Multifit pipette tips,#1,710,010,238).

P1000 filter tips (Gilson, #DF1200ST).

Moulds 15 × 15 × 5 mm (Simport Scientific, #M475–2).

#### Equipment

37 °C shaker (GFL).

Refrigerated centrifuge (Thermo Scientific, Megafuge 16R).

0.5% CO_2_ incubator (Thermo Scientific, Heracell 150i).

Biosafety 2 cabinet (Thermo Scientific, Herasafe KS).

Warm bath (MPM instruments, M418-BM).

Gilson Pipette p1000, p200, p20 and p10.

#### Media and Buffers

##### Digestion Buffer

Hyclone DMEM-F/12 1:1 (Thermo Scientific #SH30023.01) supplemented with:

10 mM HEPES (Sigma-Aldrich; #H3885),

2% BSA (Sigma-Aldrich; #A7906),

0.5 μg/mL hydrocortisone (Sigma-Aldrich; #H0135),

10 μg/mL gentamycin (Euroclone #ECM0011B; 10 mg/mL).

##### WM Medium

Hyclone DMEM-F/12 1:1 (Thermo Scientific #SH30023.01) supplemented with:

10 mM HEPES (Sigma-Aldrich; #H3885),

10 μg/mL gentamycin (Euroclone #ECM0011B; 10 mg/mL),

2 mM L- Glutamine (Euroclone ECB3000D-20; 200 mM),

1% Pennicilin/streptomycin (Euroclone #ECB3001D, 100×),

2.5 μg/mL Amphotericin B (Euroclone #ECM00 09D; 250 μg/mL).

##### TAC Buffer

1:9 of 170 mM Tris, pH 7.4 and 150 mM NH_4_Cl, pH 7.4.

##### CC Medium [6]

Hyclone DMEM-F/12 1:1 (Thermo Scientific #SH30023.01) supplemented with:

10 mM HEPES (Sigma-Aldrich; #H3885),

10 μg/mL Gentamycin (Euroclone #ECM0011B; 10 mg/mL),

2 mM L- Glutamine (Euroclone ECB3000D-20; 200 mM),

1% Pennicilin/streptomycin (Euroclone #ECB3001D, 100×),

2.5 μg/mL Amphotericin B (Euroclone #ECM00 09D; 250 μg/mL),

5 mM Nicotinammide (Sigma-Aldrich, #N0636, 250 mM).

1.25 mM N-acetylcysteine (Sigma-Aldrich, # A9165, 125 mM).

1× B27 supplement (Gibco, #17504–44, 50×).

250 ng/mL R-spondin 3 (R&D, #3500-RS/CF, 25 μg/mL)*.

5 nM Heregulin (Peprotech, #100–03, 7.14 μM)*.

5 ng/mL KGF (Peprotech, #100–19, 10 μg/mL)*.

20 ng/mL FGF10 (Peprotech, #100–26, 25 μg/mL)*.

5 ng/mL EGF (Peprotech, #AF-100-15, 5 μg/mL)*.

100 ng/mL Noggin (Peprotech, #120-10C, 20 μg/mL)*.

500 nM A83–01 (Tocris, #2939, 500 μM)*,**.

5 μM Y-27632 (Abmole,#M1817, 5 mM)*.

500 nM SB202190 (Sigma-Aldrich, #S7067, 500 μM)*.

* Add fresh the day of use.

** dissolve in DMSO.

##### Cacodylate Buffer 0.05 M pH 7.2

Prepare 100 mL of a 0.2 M stock solution of sodium cacodylate in milli Q water (4.28 g/100 ml). Then, add 8.4 mL of 0.2 M HCl to 100 mL of cacodylate stock solution, followed by the addition of milli Q water to a final volume of 400 mL. The resulting Cacodylate buffer display a molarity of 0.05 and a pH of 7.2.

#### Reagents

Collagenase enzyme stock (10×) Collagenase III (Wortington #LS004182; 2 mg/mL).

Hyaluronidase enzyme stock (100×) Hyaluronidase (Sigma #H3884; 100,000 U/mL).

70% ethanol.

TrypLE Select (Gibco #12563–029; 1×).

Matrigel Growth Factor Reduced Basement Membrane Matrix Phenol-red free (Corning #356231).

Agarose (Sigma-Aldrich, #A9539).

DMSO (Sigma-Aldrich, #D2650).

## Abbreviations

CCM: Complete culture Medium
ICH: Immunohistochemistry
PDO: Patient Derived Organoid
PDX: Patient Derived Xenograft
WM: Working Medium
